# Membrane vesicles from Red Complex bacteria: key players in oral pathogenesis, immune disruption, systemic diseases, and therapeutic insights

**DOI:** 10.3389/froh.2025.1607931

**Published:** 2025-07-28

**Authors:** Venkatramanan Mahendrarajan, Huldah Pearlin Sarah Lazarus, Gothandam Kodiveri Muthukaliannan, Sheeja Varghese, Nalini Easwaran

**Affiliations:** ^1^ School of Bio Sciences and Technology, Vellore Institute of Technology, Vellore, India; ^2^ Department of Periodontics, Saveetha Institute of Medical and Technical Sciences, Chennai, India

**Keywords:** Red Complex bacteria, outer membrane vesicles (OMVs), systemic diseases, probiotics, phytobioactives

## Abstract

The oral cavity serves as a habitat for a diverse array of microorganisms, each performing distinct functions, thereby constituting a vibrant and intricate ecological community. The most common pathogenic bacteria in the oral ecosystem are the Red Complex group, which includes *Porphyromonas gingivalis*, *Treponema denticola*, and *Tannerella forsythia*. These bacteria have several ways to inflict damage, such as creating biofilms and secreting nano-sized vesicles from their outer membrane, called Outer Membrane Vesicles (OMV). OMVs are nano structures that carry proteins, lipids, and toxins from the outer membrane of Gram-negative bacteria. The OMVs of Red Complex bacteria play a role in the onset and development of oral pathological conditions such as gingivitis and periodontitis. Additionally, a substantial body of evidence supports the notion that these OMVs may exert influence on systemic pathologies, including atherosclerosis, alzheimer's, rheumatoid arthritis, and diabetes mellitus. This review will discuss the formation and composition of Red Complex bacterial OMVs, their impacts on the oral environment, the immune response, and their correlations with various systemic diseases. The suggested treatment approach by probiotics and bioactives focuses on the genetic elements that induce the production of OMVs by the Red Complex bacteria, offering a potent means to hinder the advancement of diseases propagated through these OMVs.

## 1 Introduction

The human oral cavity, a dynamic and diverse microbial ecosystem, hosts an ever-adapting community of microorganisms. It comprises more than 800 species, including approximately 700 bacterial species, 100 fungal species, and a few viruses and protozoa (1–3). The shift in oral microbiota from a balanced commensal state to a dysbiotic state results in the progression of oral infection. Subsequently, these oral infection often gets established from dynamic interactions between microorganisms, leading to microbial colonization and complex biofilm formation (dental plaque), ultimately resulting in the exacerbation of the disease severity (4). The continued accumulation of dental plaque due to inadequate oral hygiene initiates gingival inflammation. In this microenvironment, facultative anaerobic bacteria deplete the available oxygen within the plaque biofilm, creating favorable condition for the colonization and proliferation of strict anaerobes. The inflammatory response further supports the growth of these anaerobic species by increasing the availability of host derived nutrients, thereby sustaining the dysbiotic community. This results in alterations in the bacterial composition at subgingival tissues, with an increased presence of “Red Complex” bacterial species comprising *Porphyromonas gingivalis, Tannerella forsythia*, and *Treponema denticola*—the primary etiological agents of periodontitis (5). Other contributing pathogens include Gram-positive bacteria such as *Filifactor alocis* and *Streptococcus* spp., and Gram-negative species like *Aggregatibacter actinomycetemcomitans* and *Fusobacterium nucleatum*, as well as fungal species such as *Candida* spp (6). Among the *Streptococcus* species, *S. mutans* and *S. sobrinus* have long been recognized as primary cariogenic agents, whereas *S. gordoni* was considered an accessory pathogen due to its ability to potentiate *P. gingivalis* and *A. actinomycetemcomitans* pathogenesis. The accumulation of these oral pathogens contributes to dental plaque formation, which in turn causes gingival inflammation that can progress to periodontitis in some patients. This condition is characterized by significant inflammation, bleeding, tooth loss, and occasionally pain. Pain is rarely perceived during periodontitis due to altered periodontal nociception, which is influenced by bacterial virulence factors, host immune responses, and immune suppression. This blunted pain response hampers early detection, allows disease progression, and increases the risk of systemic complications (7).

A key virulence mechanism employed by Gram negative oral pathogenic bacteria is the release of outer membrane vesicles (OMVs) which are nanoscale, bilayered structures enriched with pathogenic cargo. These OMVs, due to their small size and complex composition, have garnered considerable scientific interest. They consist of a phospholipid bilayer embedded with lipopolysaccharides (LPS), membrane proteins, and receptors on the surface (8), while their luminal contents typically include peptidoglycan fragments, periplasmic proteins, and nucleic acids such as DNA and RNA (9). The production and molecular composition of OMVs vary significantly between and within bacterial species, reflecting the tightly regulated nature of vesiculation (10).

OMVs play multifaceted roles in bacterial survival and pathogenicity. These include the removal of toxins and metabolic waste, modulation of host immune responses, horizontal gene transfer, microbial communication, biofilm development, and enhanced resistance to antimicrobial agents (11). Such functions are largely mediated by virulence determinants like LPS and proteolytic enzymes, which help the pathogen to evade innate immune defenses and establish infection (12). Periodontal diseases may further facilitate the systemic translocation of these pathogens, potentially leading to multi-organ dysfunction. Emerging evidence links oral pathogens and their OMVs to a wide range of systemic diseases, including Alzheimer's disease (13, 14) cardiovascular disease (15), diabetes mellitus (16, 17), and rheumatoid arthritis (18). These associations will be explored in detail in this review.

Although several studies have examined *P. gingivalis* OMVs in systemic diseases, a holistic analysis of the Red Complex bacterial OMV-mediated virulence, systemic impact, and immunomodulatory roles remains lacking (19–22). This review aims to bridge that gap by presenting a comprehensive evaluation of OMVs secreted by Red Complex bacteria, their associated systemic pathologies, immune interactions, and potential therapeutic strategies involving commensal microbiota and plant based bioactive compounds.

A focused literature search was conducted using the PubMed database to identify relevant studies on OMVs produced by *P. gingivalis, T. forsythia*, and *T. denticola*, particularly in the context of systemic diseases. Two keyword strategies were employed to capture both general and disease-specific research involving vesicles. The first search strategy targeted broader vesicle-related literature: (“vesicles”) AND (“Porphyromonas gingivalis” OR “Tannerella forsythia” OR “Treponema denticola”). The second, more refined search focused on vesicle types, virulence factors, and systemic disease relevance: (“outer membrane vesicles” OR OMVs OR “extracellular vesicles” OR “vesicles” OR gingipains OR “virulence factors”) AND (“Porphyromonas gingivalis” OR “Tannerella forsythia” OR “Treponema denticola”) AND (“systemic disease” OR “atherosclerosis” OR “diabetes” OR “rheumatoid arthritis” OR “alzheimer's disease” OR “neuroinflammation” OR “immune response”). Only articles published in English were considered. Peer-reviewed original research articles, reviews, and meta-analyses were included. Titles and abstracts were screened for relevance, and full texts were evaluated to ensure alignment with the study's focus on vesicle biogenesis, composition, virulence, and interactions with host systems in the context of systemic disease. Additional literature was cited to support the background, biological mechanisms, and proposed strategies of the study.

## 2 Biogenesis of OMVs from Red Complex Bacteria

*P. gingivalis* produces OMVs ranging in size from 50 to 250 nm (23). These vesicles are enriched with outer membrane proteins such as PG1823, PG2105, and PG2106, which are essential for OMV structure and function (24). Vesiculation in *P. gingivalis* is closely linked to the presence of outer membrane porins, including OmpA-like proteins (Pgm6/7) and galactose 4-epimerase (GalE). OmpA maintains membrane integrity and is involved in peptidoglycan turnover, with evidence showing its crosslinking to peptidoglycan (25). Notably, Pgm6/7 homologous to *Escherichia coli* OmpA, are critical for sustaining outer membrane architecture; their absence results in a distorted membrane and increased vesicle and gingipain production, exacerbating periodontal tissue damage and immune evasion (23). *GalE* mutants exhibit a reduction or complete loss of OMV production, while *ompA* mutants tend to overproduce OMVs, underscoring *ompA*'s regulatory role.

The expression of the major fimbrial subunit FimA correlates positively with vesiculation. OMV production is significantly reduced in *fimA* and *fimR* mutants, suggesting that the Fim locus plays a regulatory role in vesicle release, adhesion, host invasion, and pathogenicity (26). *P. gingivalis* contains two types of O-antigens: negatively charged LPS (A-LPS) and neutral LPS (O-LPS) (24). A-LPS, composed of phosphorylated mannan repeats linked to the lipid A core, which is integral to OMV formation. Dephosphorylation of A-LPS by outer membrane protein PG0027 destabilizes the membrane, facilitating vesicle release (23), which also modulates gingipain production. Mutants lacking A-LPS exhibit reduced levels of OMV-associated gingipains (27), and instead show increased levels of envelope proteins like RagA and RagB, typically absent in wild-type OMVs. Silencing the *rgpA* gene, which encodes arginine-specific gingipain A, leads to a reduction in OMV production (23). Key virulence factors—including arg-gingipains (Rgp), lys-gingipains (Kgp), and hemagglutinins (Hag)—are secreted via the Por secretion system (PorSS). Their C-terminal domain (CTD) ensures proper localization to the bacterial surface and is glycosylated with A-LPS, reinforcing the centrality of A-LPS in OMV biogenesis and virulence. Charged LPS microdomains, highly concentrated on the *P. gingivalis* outer surface, influence OMV formation. Deacylation of anionic LPS promotes membrane curvature, enhancing vesiculation (24).

In *T. forsythia*, the S-layer proteins TfsA and TfsB mediate adhesion to gingival cells and hemagglutination. While the native S-layer maintains a 1:1 TfsA:TfsB ratio, OMVs display a 3:1 ratio due to irregular S-layer formation and protein loss. Given the complexity of forming curved S-layers, this shift may aid OMV production by enhancing curvature (28). Beyond structural components, environmental stressors such as heat, nutrient deprivation (e.g., lysine deficiency), sublethal antibiotic exposure, oxidative damage, hemin limitation, and serum addition can trigger hypervesiculation without causing cell lysis. These stimuli, including quorum sensing and envelope stress, induce stress-responsive vesicle overproduction.

While OMV biogenesis has been extensively studied in other Red Complex bacteria such as *Porphyromonas gingivalis*, such mechanistic insights are notably absent for *T. denticola*. Most available literature on *T. denticola* OMVs—focuses on structural, proteomic, and virulence-related characterization rather than the molecular process of biogenesis.

## 3 Components of Red Complex bacterial OMVs

Bacterial OMVs serve as multifunctional tools to enhance microbial survival and pathogenesis. They transport virulence factors, mediate immune modulation, and promote bacterial communication (29). Structurally, OMVs possess a lipid bilayer rich in outer membrane proteins and LPS, while the lumen contains periplasmic proteins, enzymes, lipids, and nucleic acids, including DNA and RNA (30).

Among the critical OMV components in *P. gingivalis*, gingipains represent major virulence factors. The gene PGN_1251 (*gtfB*), involved in gingipain processing, encodes a protein homologous to glycosyltransferase-1. *gtfB* mutants lacking both O-LPS and A-LPS, show diminished surface expression of gingipains and other key proteins, underscoring its role in maintaining OMV structural integrity (23). The C-terminal domain (CTD) domain, about 70 amino acids in length is essential for surface attachment and secretion of OMV-associated proteins, including gingipains. Notably, OMV composition varies among *P. gingivalis* strains. For instance, fimbriated strains contain FimC, FimD, and FimE in their OMVs, which are absent in non-fimbriated strains (23).

OMVs from *T. forsythia* are highly heterogeneous, containing at least 297 proteins. Approximately 31% are substrates of the type IX secretion system, with 54% localized to the membrane, 12% to the lumen, and the remainder of unknown localization. TonB-dependent receptors (TDRs) constitute 70% of vesicle membrane proteins. Key proteins include: N-protein (Tanf_02375), involved in DNA binding and helicase activity, PorP (Tanf_02355), a DNA-binding auxiliary protein, proteinases with CTD-like domains, S8 peptidase (Tanf_00440, mirolase) with serine endopeptidase activity and Karilysin (Tanf_06550), a metalloendopeptidase (28). Approximately 98 lipoproteins were predicted in *T. forsythia* OMVs, including membrane-bound enzymes such as glucosyl hydrolases, peptidyl-prolyl cis-trans isomerase, and HmuY. Vesicle lumens contain sialidases, glucosidases, and 12 predicted glycosyl hydrolases such as galactosidases, mannosidases, and a newly identified membrane-associated fucosidase (28, 31). These findings, largely based on *in silico* predictions, require further experimental validation.

Using label-free quantitative proteomics, 1,448 proteins were identified from *T. denticola*, including 90 outer membrane proteins such as lipoproteins and β-barrel proteins. Distinct signal cleavage patterns differentiated outer membrane from inner membrane lipoproteins. OMVs were enriched in virulence factors like dentilisin, dentisilin protease complex PrtP, its auxiliary lipoproteins PcrA and PcrB and FGE-sulfatase domain-containing lipoproteins. Native PAGE revealed large protein complexes, including major surface protein (Msp) and β-barrel proteins. Homologous analysis also uncovered new outer membrane proteins in *T. pallidum*, offering promising vaccine targets for both pathogens (28, 32). Additional components include LrrA (TDE2258), a leucine-rich repeat surface antigen, and BspA, a putative lipoprotein implicated in interkingdom communication, host colonization, and coaggregation with other oral bacteria (28).

## 4 Red Complex bacterial OMVs and periodontal disease

Migration of Red Complex bacteria and their byproducts including LPS and proinflammatory cytokines, into distal organs through the bloodstream results in systemic diseases, often originating from plaque accumulation in the oral cavity (33, 34). The resulting polymicrobial biofilm forms through coordinated interactions among diverse bacteria, mediated by adhesins and receptors (35). *P. gingivalis*, a key member of the Red Complex, is extensively studied in oral infectious diseases. Notably, infection with *P. gingivalis* alone is sufficient to induce periodontitis in non-human primates, highlighting its pathogenic potential (36). OMVs from *P. gingivalis* facilitate interspecies aggregation, such as between *Staphylococcus aureus*, *Streptococcus* spp., and *Candida* spp (37). OMVs also promote interactions between *T. denticola* and *Lactobacillus laburnum*, enabling motility for typically non-motile bacteria within the oral microenvironment (38). Two adhesins, FimA and Mfa, present on *P. gingivalis* OMVs, enhance bacterial attachment, while OMV-associated gingipains inhibit the biofilm formation of commensal species, favoring colonization by pathogenic bacteria (39). However, the specific mechanisms underlying *P. gingivalis* OMV-mediated biofilm formation remain unexplored (36).

*T. denticola* OMVs are enriched with dentilisin, a trypsin-like protease complex that degrades extracellular matrix components such as fibronectin and laminin, thereby weakening connective tissue architecture (40). Additionally, OMVs contain the Msp, which disrupts epithelial barrier function and alters cell adhesion, facilitating bacterial invasion into deeper gingival tissues (41). These vesicles also contribute to biofilm maturation and enhance the cooperative virulence of the Red Complex consortium. Additionally, *T. denticola* OMVs are involved in interspecies and host cell communication, contributing to robust biofilm development, a major factor in chronic periodontitis (42).

*T. forsythia* OMVs similarly carry potent proteases such as karilysin and mirolase, which break down ECM proteins and basement membrane structures, promoting detachment of the junctional epithelium and periodontal pocket formation (31). The OMV-associated S-layer glycoproteins further assist in colonization and persistence in the subgingival environment (43). The OMVs also promote sialic acid catabolism and associated biofilm formation, aggravating periodontal disease severity (44). By enabling deeper bacterial penetration and local tissue degradation, OMVs from these pathogens play a direct role in the structural breakdown of periodontal tissues characteristic of periodontitis.

## 5 Red Complex bacterial OMVs and host cell interactions

OMVs derived from *P. gingivalis* are internalized by gingival epithelial and endothelial cells through two primary mechanisms: actin-mediated endocytosis and lipid raft dependent pathways. The former involves OMV recognition of α5β1 integrin on host cells, triggering F-actin polymerization regulated by phosphatidylinositol 3-kinase (PI3K). The latter depends on fimbriae and engages PI3K, Rac1, and regulatory GTPases (45). The route of internalization is OMV size dependent. OMV uptake disrupts key signaling molecules, including transferrin receptor (TfR) and paxillin/focal adhesion kinase (FAK), impairing epithelial cell motility (46, 47). *P. gingivalis* OMVs also interfere with oral squamous cells via gingipains (24, 48) and inhibit fibroblast and endothelial cell proliferation, hindering angiogenesis and delaying wound healing in periodontal tissues (49). These vesicles reduce endothelial nitric oxide synthase (eNOS) expression, leading to oxidative stress. Moreover, OMVs trigger apoptosis, cellular activation, and cytokine secretion by activating host pattern recognition receptors (PRRs) (50). The peptidoglycan components of *P. gingivalis* OMVs stimulate autophagy (51), while macrophages exposed to OMVs release pro- and anti-inflammatory cytokines, including interleukins IL-6, IL-10, IL-12p70, interferon-β (IFN-β), and tumor necrosis factor-α (TNF-α), promoting glycolysis-mediated apoptosis (52). Nuclear factor kappa B (NF-*κ*B) activation further modulates monocyte and macrophage responses. Furthermore, *P. gingivalis* OMVs, through LPS and Toll-like receptor 2 (TLR2) interactions, promote osteoclast differentiation, contributing to alveolar bone resorption. Intriguingly, these vesicles can also stimulate the invasion and migration of oral squamous cell carcinoma, suggesting a role in tumor progression (53, 54).

*T. denticola* OMVs, rich in the protease dentilisin, degrade intercellular adhesion proteins, enhancing bacterial invasion. Additional components such as Msp and chymotrypsin-like proteases contribute to cytotoxicity by forming pores in epithelial cells (44). *T. denticola* OMVs also contain LPS, which is cytotoxic to gingival epithelium. These effects collectively lead to extracellular matrix degradation and chronic periodontitis (55).

Recent studies have demonstrated that OMVs of *T. forsythia* play a significant role in host–pathogen interactions within the oral cavity. These vesicles are capable of adhering to and being internalized by human oral epithelial cells through mechanisms mediated by surface virulence factors such as the BspA protein (43). Upon internalization, *T. forsythia* OMVs have been shown to stimulate pro-inflammatory responses, including the secretion of interleukin-8 (IL-8) and TNF-α, via TLR2-dependent signaling pathways (56). Proteomic analyses of *T. forsythia* OMVs further support their involvement in host modulation, revealing the presence of multiple glycosidases, proteases, and autotransporters capable of degrading host extracellular matrix components and altering immune responses (56). These findings suggest that *T. forsythia* OMVs contribute actively to periodontal pathogenesis by mediating epithelial cell responses and facilitating tissue destruction.

## 6 OMVs from Red Complex bacteria and systemic diseases

### 6.1 Atherosclerosis

Atherosclerosis, a condition characterized by arterial plaque buildup and reduced vessel elasticity, significantly increases the risk of cardiovascular events. There is accumulating evidence linking oral bacterial infections, particularly those involving biofilm-forming pathogens, with atherosclerosis. *P. gingivalis* is frequently detected in individuals with vascular disease, yet the precise mechanisms remain under investigation (57–59). OMVs are nanostructures capable of traversing vascular membranes more efficiently than whole bacteria (60, 61). They can influence key factors such as blood coagulation, endothelial integrity, calcium deposition, and lipoprotein metabolism. Among oral pathogens, *P. gingivalis, T. forsythia*, and *T. denticola* have been implicated in cardiovascular pathology (62, 63).

*P. gingivalis* OMVs in the bloodstream have been shown to promote platelet aggregation, a crucial step in thrombus and plaque formation (64). They also enhance foam cell development by increasing low-density lipoprotein (LDL) accumulation in macrophages, fueling atherosclerotic progression (65). Additionally, OMVs suppress eNOS, a key vasoprotective enzyme, via Rho-associated kinase (ROCK) activity. This suppression is mediated by the extracellular signal-regulated kinase (ERK1/2) and mitogen-activated protein kinase (MAPK) pathways and can be reversed with ROCK inhibitors like Y-27632 (66). *In vivo* studies using zebrafish larvae models have shown increased vascular damage upon *P. gingivalis* OMV exposure (67). Gingipains on OMV surfaces degrade platelet endothelial cell adhesion molecule-1 (PECAM-1 or CD31), a protein essential for endothelial junction integrity. Reduced PECAM-1 levels increase vascular permeability, exacerbating vascular inflammation and lesion development. OMVs also activate the ERK1/2 and runt-related transcription factor 2 (RUNX2) signaling pathways in vascular smooth muscle cells, promoting calcification and accelerating atherosclerosis (68).

**Figure 1:**
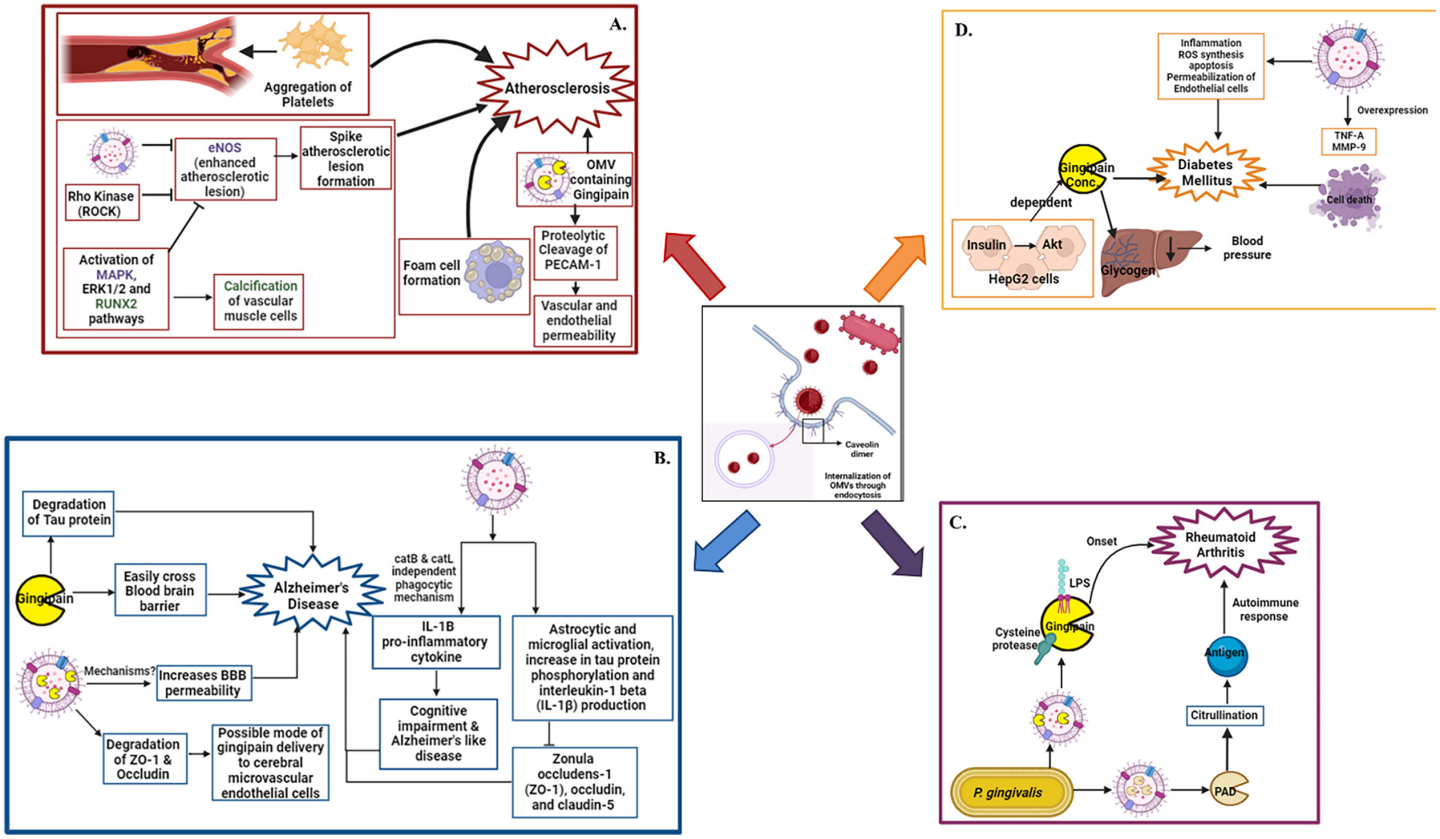
Oral pathogen OMV mediated systemic diseases: internalization of OMVs in host tissues through endocytosis. After internalization OMVs interact with various pathways and leads to emergence of various systemic diseases. The disease onset and its interlinking pathways are represented in panel **(A)**. Atherosclerosis, **(B)** Alzheimer's disease, **(C)** Rheumatoid arthritis, **(D)** Diabetes mellitus respectively. Figure created with BioRender.com.

*T. forsythia* OMVs and other bacterial components, were shown to stimulate foam cell formation in macrophages and accelerate lesion development in ApoE^−^/^−^ mice via its BspA protein and OMV associated molecules. Chronic infection in these models also caused dyslipidemia, elevated serum amyloid A, and reduced nitric oxide, hallmark indicators of atherosclerotic risk. In human studies, *T. forsythia* DNA and virulence genes, including those associated with OMV cargo, have been detected in atherosclerotic plaques, alongside *T. denticola* and *P. gingivalis*, reinforcing a mechanistic link. *T. denticola* can activate endothelial cells by inducing the expression of IL8 and monocyte chemoattractant protein-1 (MCP1) which can facilitate chemotaxis and aggregation of monocytes which might cause atherosclerosis (69). The detailed summary were represented in Figure 1.

### 6.2 Alzheimer's disease

*P. gingivalis* and its toxic protease, gingipain, have been identified in the brains of patients with Alzheimer's disease (AD) (45). A growing body of evidence suggests that bacterial OMVs can enhance blood–brain barrier (BBB) permeability, although the exact mechanisms remain unclear (45). Despite the brain being an inhospitable environment for anaerobic bacteria such as *P. gingivalis*, its DNA and LPS have been detected in AD patient brain tissue, likely due to the nano-scale size of OMVs, which can more readily cross the BBB (70, 71). The “Gingipains Hypothesis” and Pg-induced iron dyshomeostasis, alongside reduced salivary lactoferrin and cholinergic dysfunction, suggest a broader pathogenic role. Pg's Type IX secretion system enables the release of toxin-rich OMVs, offering a more plausible explanation for widespread toxin distribution in the brain. This model unifies microbial and neurodegenerative hypotheses, providing a comprehensive framework for understanding and managing sporadic AD (72). Gingipains present in *P. gingivalis* OMVs have been shown to degrade tight junction proteins such as zonula occludens-1 (ZO-1) and occludin in human cerebral microvascular endothelial cells (hCMEC/D3), suggesting a possible route of entry into the brain (73, 74). Moreover, OMVs stimulate the production and maturation of IL-1β, a pro-inflammatory cytokine, via cathepsin B- and L-independent phagocytic mechanisms. These immune responses have been linked to cognitive decline and Alzheimer-like symptoms in murine models (75).

One study reported that repeated abdominal injections of *P. gingivalis* OMVs over 12 weeks led to their translocation into mouse brains (76). Another investigation found that OMVs were detectable in cortical and hippocampal regions within just three days of oral administration (77). Their presence was associated with astrocyte and microglial activation, increased phosphorylation of tau protein, and elevated hippocampal IL-1β levels. These OMVs also reduced expression of tight junction proteins, including ZO-1, occludin, and claudin-5 factors crucial for maintaining BBB integrity and cognitive function (77). Tau protein, predominantly found in neurons, plays a vital role in stabilizing microtubules. Gingipains in OMVs are highly neurotoxic, both *in vitro* and *in vivo*, and disrupt tau homeostasis, contributing to the pathogenesis of AD (78). Additionally, gingival exposure to *P. gingivalis* or its OMVs induces periodontitis, systemic inflammation, and neuroinflammatory responses in murine models, leading to memory impairment-like behaviors. OMVs were detected in the trigeminal ganglia and hippocampus, indicating a potential neural route of brain entry and subsequent impact on AD pathology (79). These mechanisms are summarized in Figure 1.

### 6.3 Rheumatoid arthritis

Rheumatoid arthritis (RA) is a chronic autoimmune disease characterized by systemic inflammation, primarily targeting the joints. *P. gingivalis* contributes to RA pathogenesis not only by affecting the periodontium but also by initiating protein citrullination in synovial tissues and neurons. The bacterium facilitates citrullination via proteolytic cleavage of arginine-containing peptides, a process central to autoantigen formation in RA (80). Inactivation of *P. gingivalis* arginine-specific gingipains significantly reduces citrullination. OMVs are believed to be a major delivery system for gingipains, directly linking them to RA progression. Citrullination occurs after gingipain-mediated cleavage, through the action of peptidyl arginine deiminase (PAD), an enzyme enriched in *P. gingivalis* OMVs. Deletion of the PAD gene abolishes citrullination activity entirely (81).

When incubated with host proteins such as fibrinogen and α-enolase, *P. gingivalis* induces both protein degradation and citrullination, leading to the formation of neoantigens. These altered proteins provoke autoimmune responses, including the generation of anti-citrullinated protein antibodies (ACPAs), which are present in approximately 75% of RA patients (82, 83). ACPAs can be detected up to a decade before clinical onset, and their presence is strongly correlated with periodontitis (84, 85) Thus, OMVs from *P. gingivalis*, enriched with LPS, gingipains, and PAD, play a key role in driving citrullination and the subsequent autoimmune cascade in RA. The underlying mechanisms are illustrated in Figure 1.

### 6.4 Diabetes mellitus

Emerging research implicates OMVs from oral pathogens in the pathogenesis of diabetes mellitus, a metabolic disorder characterized by chronic hyperglycemia and insulin resistance (86). This relationship appears to be bidirectional: diabetes increases susceptibility to periodontal infections and impairs wound healing, while OMVs from pathogens like *P. gingivalis* exacerbate systemic inflammation and metabolic dysfunction (87). In murine models, *P. gingivalis* OMVs deliver gingipains to the liver, impairing insulin-stimulated glycogen synthesis and consequently elevating blood glucose levels. In hepatic HepG2 cells, insulin activates the Akt/glycogen synthase kinase-3β pathway, which is disrupted in a gingipain concentration–dependent manner (86). Moreover, OMVs contribute to diabetic complications such as retinopathy. *In vitro* studies have shown that *P. gingivalis* OMVs compromise the integrity of the retinal blood barrier by inducing inflammation, reactive oxygen species (ROS) production, apoptosis, and endothelial permeability. They also upregulate TNF-α and matrix metalloproteinase-9 (MMP-9), leading to retinal endothelial cell damage and death (88). The summary were represented in Figure 1.

## 7 Immune responses mediated by OMVs of Red Complex bacteria

Immunoglobulin G (IgG) antibodies from *P. gingivalis* seropositive patients interact strongly with wild-type *P. gingivalis*, but only modestly with OMV-deficient mutants. OMVs from *P. gingivalis* serve as reservoirs of immunogenic antigens and active proteases that contribute to tissue degradation in periodontal disease (60). These vesicles induce cellular activation, cytokine production, and apoptotic cell death by enhancing the activity of PRRs in gingival epithelial cells. Additionally, OMVs engage with macrophages via PRRs, triggering the secretion of both pro- and anti-inflammatory cytokines, thereby sustaining chronic inflammation (89).

Periodontitis is characterized by the activation of various immune cells including neutrophils, B and T lymphocytes, natural killer cells, macrophages, and osteoclasts, primarily driven by pro-inflammatory cytokines. When exposed to *T. forsythia* OMVs, periodontal ligament fibroblasts express significantly higher levels of IL-6, IL-8, and MCP-1 than when exposed to the bacteria themselves. These chemokines, particularly IL-8 and MCP-1, recruit neutrophils to the site of inflammation (31). OMVs from *P. gingivalis* also induce foam cell formation, which is associated with the release of inducible nitric oxide synthase (iNOS) and nitric oxide (NO). Moreover, *P. gingivalis* OMVs stimulate IL-8 secretion, while lipooligosaccharide (LOS) and *T. denticola* OMVs elicits strong inflammatory responses in human gingival fibroblasts via IL-6, IL-8, MCP-1, prostaglandin C, and NO production (23). Persistent overproduction of these mediators can result in significant tissue destruction. *P. gingivalis* OMVs contribute to periodontitis by delivering sRNA45033, which targets CBX5 in human periodontal ligament cells, leading to apoptosis and inflammatory cytokine release. CBX5 modulates p53 DNA methylation, revealing a novel OMV-mediated host–pathogen interaction in periodontal disease (90).

Gingipains present on *P. gingivalis* OMVs degrade IgG, immunoglobulin M (IgM), and complement component C3, compromising the protective function of serum (48, 91) OMVs from *P. gingivalis*, *T. forsythia*, and *T. denticola* interact with PRRs on monocytes and macrophages, leading to hypersecretion of NF-*κ*B, TNF-α, IL-1β, and IL-8 (55). Specifically, *P. gingivalis* OMVs trigger strong TLR2 and TLR4 responses, along with moderate activation of nucleotide-binding oligomerization domain-containing proteins NOD1 and NOD2, and other TLRs (TLR4, TLR7, and TLR8), whereas OMVs from the other two Red Complex members provoke much weaker responses (92).

Furthermore, OMVs from *P. gingivalis* impair the expression of leukocyte surface markers on human umbilical vascular endothelial cells, thereby disrupting major histocompatibility complex class II (MHC II)-mediated adaptive immune responses (93). These OMVs also activate human neutrophils through a surface-coating mechanism that enhances degranulation and inhibits neutrophil apoptosis. Notably, gingipain proteases within OMVs degrade antimicrobial peptides such as LL-37 and enzymes like myeloperoxidase from neutrophil granules (94). This immune evasion strategy is similarly employed by *A. actinomycetemcomitans*, reinforcing the role of OMVs in counteracting neutrophil-mediated antimicrobial activity.

OMVs from *P. gingivalis* and *T. forsythia* promote osteoclast differentiation by activating TLR2 signaling in osteoclast precursors. Their osteoclastogenic effects are mediated by lipoproteins and LPS, as shown by reduced activity following treatment with lipoprotein lipase and polymyxin B (95). Infection of macrophages with *T. forsythia* results in the release of two distinct EV populations: macrophage-derived EVs enriched with pro-inflammatory mediators and *T. forsythia*-derived OMVs carrying virulence factors like BspA and GroEL. These OMVs trigger TLR2-mediated inflammatory responses and are actively released in response to macrophage-derived signals, highlighting their role in periodontitis progression (56).

**Figure 2:**
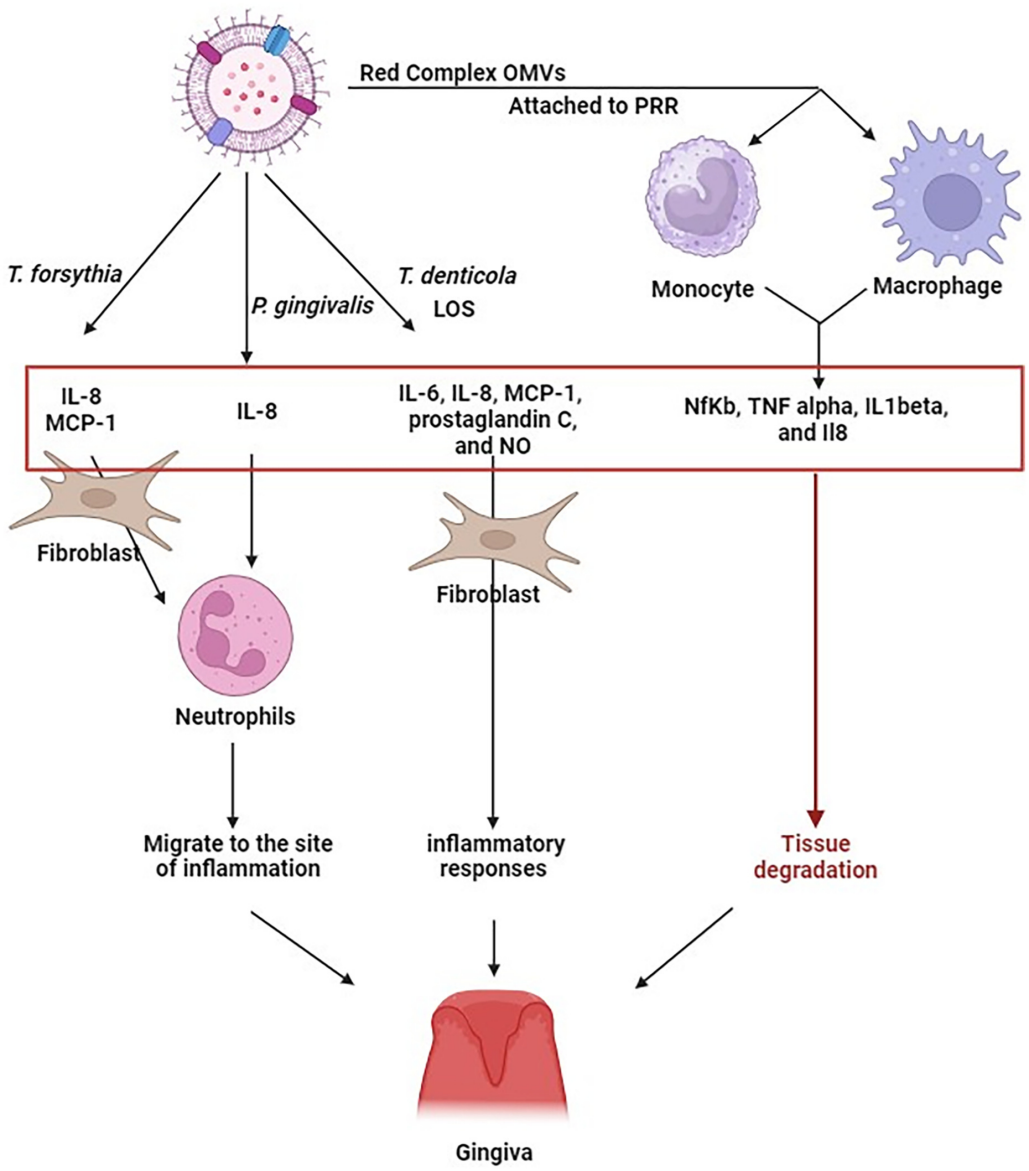
Exposure of periodontal ligament fibroblasts to *T. forsythia* OMV, leads to an increased production of IL-6, IL-8, and MCP-1. OMVs from *P. gingivalis* can induce the secretion of IL-8. Similarly, OMVs from *T. denticola* carrying lipooligosaccharides, produced IL-6, IL-8, monocyte chemoattractant protein-1 (MCP-1), prostaglandin E2 (PGE2), and nitric oxide (NO) in human gingival fibroblasts. The presence of IL-8 and MCP-1, in particular, triggers the migration of neutrophils towards the inflammation site. OMVs from *P. gingivalis*, *T. forsythia*, and *T. denticola*, upon interacting with monocyte and macrophage receptors, stimulate hypersecretion of NfKb, TNF alpha, IL1beta, and IL8. The continuous generation of these cytokines may potentially exacerbate deterioration of tissue. This indicates that the immune response to Red Complex OMVs might be more intense than to the bacteria themselves. Figure created with BioRender.com.

Moreover, *P. gingivalis* OMVs modulate cellular metabolism in human trophoblast cells by disrupting glycolysis and suppressing ROS production. While overall cell viability remains unaffected, cellular migration and invasion are diminished, leading to a quiescent state. In murine models, OMV exposure results in reduced expression of glucose transporter 1 (GLUT1) and glycolytic activity, ultimately decreasing fetal weight gain (96). The overall representations were given in Figure 2.

## 8 Strategies for the prevention of Red Complex bacterial OMV-mediated systemic diseases

The oral microbiota is a diverse and dynamic ecosystem comprising bacteria, fungi, viruses, and protozoa that colonize various niches of the oral cavity, including the teeth, tongue, gingiva, and mucosa. This complex microbial community plays a vital role in maintaining oral health and protecting against pathogenic infections (97). A key function of the commensal oral microbiota is “colonization resistance”, the prevention of pathogen adherence and proliferation through competition for binding sites and essential nutrients, or through direct microbial antagonism (98). Moreover, the normal oral flora can modulate host immune responses by inducing antibody and cytokine production, thereby enhancing host defense mechanisms against pathogens. Probiotic and prebiotic therapies have been proposed as promising strategies to restore or enhance this protective microbial balance (99).

Species of *Lactobacillus*, including *L. acidophilus*, *L. casei, L. paracasei, L. plantarum, L. rhamnosus*, and *L. salivarius*, have demonstrated significant antibacterial activity against *P. gingivalis*, a keystone member of the Red Complex consortium (100–103) Other species such as *L. salicinius, L. rhamnosus,* and *L. paracasei* exhibit antimicrobial effects against multiple oral pathogens—including *Streptococcus mutans*, *P. gingivalis*, *Fusobacterium nucleatum*, and *A. actinomycetemcomitans*—even in heat-killed forms, suggesting the presence of heat-stable antimicrobial compounds (104).

Supernatants from several *Lactobacillus* species, such as *L. acidophilus*, *L. casei, L. crispatus, L. fermentum, L. plantarum, L. rhamnosus, L. salivarius*, and *L. vaginalis*, have shown broad-spectrum antimicrobial activity against Gram-negative *A. actinomycetemcomitans* and Gram-positive *Actinomyces naeslundii* (105–109)*.* Notably, the supernatant of *L. kefiranofaciens* inhibited *S. mutans* biofilm formation both phenotypically and at the transcriptomic level (110). These effects suggest that *Lactobacillus* species exert their protective role through competitive exclusion, nutrient limitation, and secretion of extracellular antimicrobial metabolites.

As previously discussed, *P. gingivalis* OMV production can be downregulated through mutations in key genes such as *fimA, fimR*, and *rgpA*. Targeting these genes presents a viable strategy for mitigating OMV-mediated pathogenesis. Supporting this hypothesis, supernatants from *L. rhamnosus, L. acidophilus*, and *L. reuteri* have been shown to downregulate *fimA* and *rgpA* expression in *P. gingivalis* (111)*.* A study by Yang KM et al. further confirmed the inhibition of *rgpA* by *L. reuteri* supernatant (106). These findings suggest that Lactobacillus-derived probiotics, and potentially their secreted metabolites or vesicles, may be developed as therapeutic agents targeting OMV biogenesis in Red Complex pathogens. However, robust *in vitro* and *in vivo* studies are necessary to validate these approaches and fully explore their clinical potential (97).

In addition to probiotics, natural bioactive compounds derived from plants have shown promise in promoting oral health and targeting the pathogenic mechanisms of Red Complex bacteria (112). Among these, curcumin, a polyphenolic compound extensively studied for its anti-inflammatory and antimicrobial properties, has demonstrated significant inhibitory effects on the proliferation of *P. gingivalis*, *T. forsythia*, and *T. denticola* (113).

Curcumin reduces the cytotoxic effects of *P. gingivalis* OMVs by inhibiting OMV-induced epithelial cell apoptosis and preventing bacterial attachment. It also downregulates pro-inflammatory genes such as IL-6, IL-1β, and TNF-α in human epithelial cells (114). Importantly, curcumin has been shown to suppress the expression of key OMV-associated genes, *fimA* and *rgpA*, thereby reducing OMV production and virulence (115). Other plant-derived bioactives exhibit similar mechanisms. Resveratrol, a natural phenolic compound, significantly inhibits *fimA* and *rgpA* expression in *P. gingivalis* (116). Epigallocatechin-3-gallate (EGCG), a major catechin in green tea, also downregulates these genes (117). Rhein, an anthraquinone compound, markedly reduces their expression as well (118). Coumarin has been shown not only to suppress *rgpA* expression but also to reduce overall pathogenicity of *P. gingivalis* (119).

**Figure 3:**
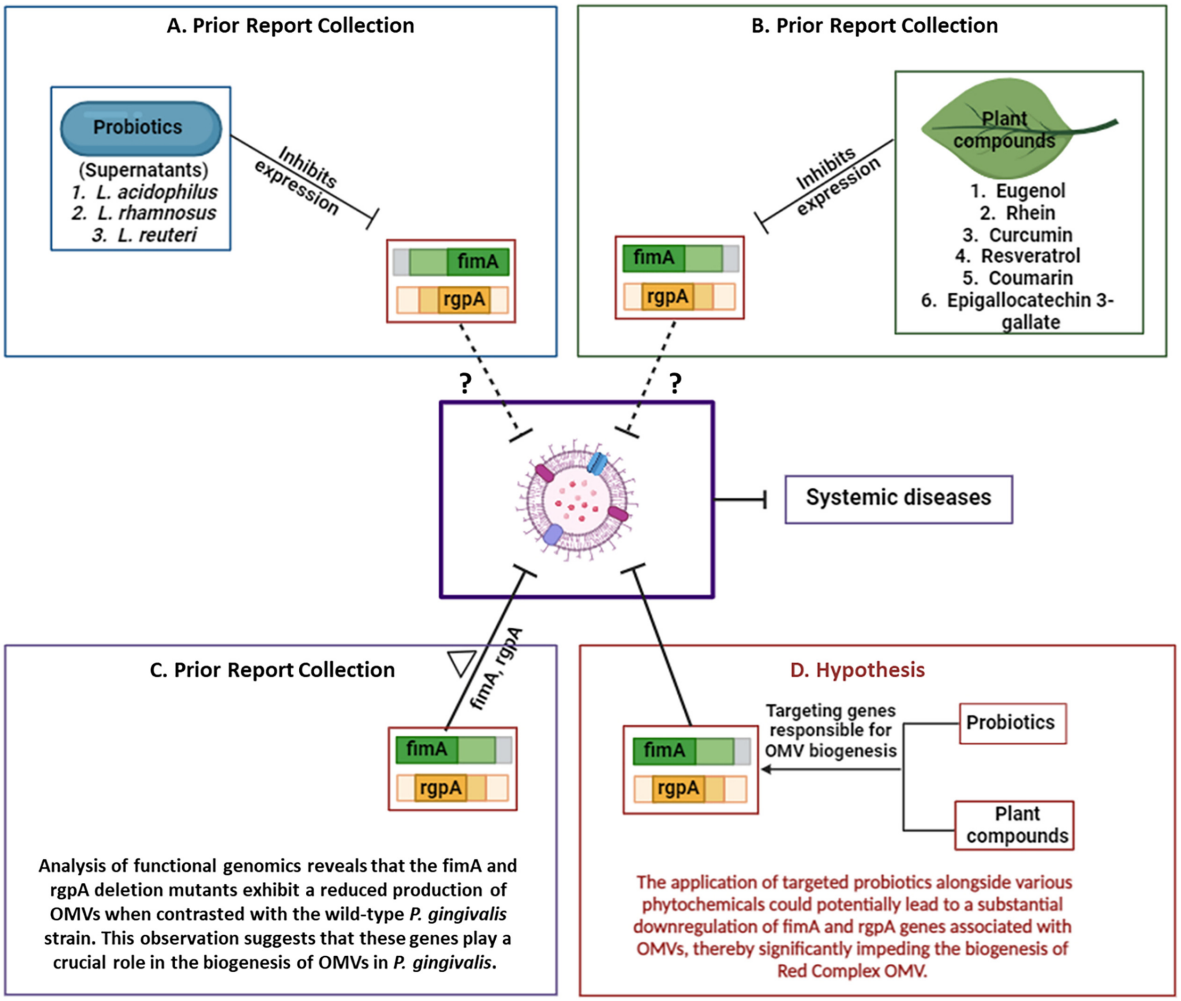
The proposed therapeutic strategy combining *Lactobacillus* and natural products against the OMVs of Red complex pathogens. **(A)** Probiotic strains and **(B)** plant-derived compounds have been shown to inhibit the expression of fimA and rgpA genes. **(C)** Deletion of these genes significantly impairs OMV production in P. gingivalis. **(D)** Based on these findings, we hypothesize that targeting fimA and rgpA using probiotics and phytochemicals may reduce OMV release and consequently mitigate the progression of OMV associated systemic diseases. Figure created with BioRender.com.

Further, omega-3 fatty acids such as docosahexaenoic acid (DHA) and eicosapentaenoic acid (EPA) significantly inhibit *rgpA* gene expression, as demonstrated by Sun et al. (120). Eugenol, a phenolic compound commonly found in clove oil, also suppresses both *fimA* and *rgpA* expression (121). Collectively, these natural compounds offer a novel therapeutic avenue by attenuating OMV production at the genetic level, thereby weakening the virulence and disease-promoting capacity of Red Complex pathogens. The therapeutic strategies were represented in Figure 3.

## 9 Discussion

OMVs released by red complex bacteria emerge as pivotal agents in both localized periodontal pathology and systemic inflammatory diseases (30). This review consolidates evidence that substantiates OMVs as not merely byproducts of microbial metabolism, but as sophisticated vectors of virulence capable of modulation of host immunity, alteration of epithelial integrity, and facilitating interspecies microbial synergy. The multifactorial composition of OMVs, including gingipains, LPS, fimbrial proteins, and various secretion system substrates, reflects the evolved mechanisms these bacteria employ to subvert host defenses (20). The presence of gingipains within OMVs has been particularly emphasized for their proteolytic degradation of immunoglobulins (IgG, IgM), complement proteins (C3), and junctional proteins such as PECAM-1 and tight junction proteins (ZO-1, occludin), all of which are critical for maintaining immune and endothelial barrier functions (74).

The internalization of OMVs by host cells through actin-mediated or lipid raft–dependent endocytosis initiates a cascade of detrimental effects, including impaired wound healing, oxidative stress, pro-inflammatory cytokine secretion, and even the promotion of oncogenic behavior in oral squamous cells. In *P. gingivalis*, for instance, gingipains contained in OMVs not only contribute to periodontal tissue destruction but also promote osteoclastogenesis through TLR2 signaling, thereby accelerating alveolar bone loss (20). In atherosclerosis, OMVs from *P. gingivalis* have been shown to induce foam cell formation (65), promote platelet aggregation, suppress eNOS, and activate RUNX2-mediated vascular calcification, through well-characterized ROCK and MAPK signaling pathways (66). Similarly, OMVs have been implicated in the pathogenesis of Alzheimer's disease by breaching the BBB, degrading tight junction proteins, and triggering neuroinflammation and tau phosphorylation. The contribution of OMVs to rheumatoid arthritis is likewise strongly supported wherein *P. gingivalis* OMVs, enriched with gingipains and PAD, mediate citrullination of host proteins, generating neoantigens and driving the production of ACPAs, a hallmark of RA pathogenesis (81). In diabetes mellitus, OMVs impair insulin signaling and hepatic glycogen synthesis through gingipain-mediated disruption of the Akt/GSK-3β pathway (86). These vesicles further exacerbate diabetic complications such as retinopathy by promoting oxidative stress, apoptosis, and increased endothelial permeability.

A recurring theme across these disease models is the heightened immunostimulatory potential of OMVs compared to whole bacteria. Periodontal ligament fibroblasts and macrophages exhibit exaggerated inflammatory responses, including elevated IL-6, IL-8, TNF-α, and NO production, upon exposure to OMVs (52). This hyperactivation suggests that OMVs may act as amplifiers of immune-mediated tissue damage. Importantly, therapeutic modulation of OMV biogenesis and function represents a promising avenue. Several *Lactobacillus* strains, including *L. rhamnosus*, *L. acidophilus*, and *L. reuteri*, have been demonstrated to downregulate OMV-associated genes such as *fimA* and *rgpA* in *P. gingivalis*, indicating a role in mitigating vesicle-mediated pathogenesis. Natural bioactives like curcumin, resveratrol, and EGCG also exhibit gene-suppressive effects on these virulence factors, further supporting the potential of probiotic and phytotherapeutic strategies. Collectively, the data presented support a paradigm in which Red Complex bacterial OMVs function as highly effective virulence delivery systems, influencing not only periodontal disease progression but also distant systemic pathologies. Future studies should prioritize *in vivo* validation of OMV-targeting therapies and elucidate the full spectrum of OMV-host interactions, including their epigenetic and metabolic effects.

## 10 Conclusion

Red Complex bacteria are key contributors to periodontal diseases and are strongly associated with systemic conditions such as atherosclerosis, alzheimer's disease, rheumatoid arthritis, and diabetes mellitus. As highlighted in this review, OMVs are critical virulence factors of these pathogens. Probiotic approaches, particularly involving *Lactobacillus* species and their secreted products, have shown potential in downregulating OMV-associated genes, including *fimA* and *rgpA* in these pathogens. Similarly, plant-derived bioactive compounds such as curcumin, resveratrol, and EGCG have demonstrated promising effects in suppressing the genetic machinery responsible for OMV biogenesis. Despite these encouraging findings, there remains a significant gap in the direct study of OMV biogenesis inhibition in the Red Complex bacteria. Future research focused on identifying and targeting genes involved in vesicle production could provide innovative strategies for the prevention and treatment of Red Complex bacterial oral and systemic diseases.
